# 5-Phenyl-2-(4-pyrid­yl)pyrimidine

**DOI:** 10.1107/S160053680800398X

**Published:** 2008-02-13

**Authors:** Marie-Pierre C. Santoni, Siu Hong Yu, Garry S. Hanan, Anna Proust, Bernold Hasenknopf

**Affiliations:** a Département de Chimie, Université de Montréal, CP 6128, Succ. Centre-ville, Montréal, Québec, Canada H3C 3J7; b Laboratoire de Chimie Inorganique et Matériaux Moléculaires, UMR 7071, Université Pierre et Marie Curie, Paris, France

## Abstract

The title compound, C_15_H_11_N_3_, crystallizes with two independent mol­ecules in the asymmetric unit. The dihedral angles between the phenyl and pyridine rings in each mol­ecule are 53.48 (5) and 50.80 (5)°. In the crystal structure, weak inter­molecular C—H⋯N hydrogen bonds connect mol­ecules into one-dimensional chains. In addition, the crystal structure is stabilized by weak C—H⋯π(arene) inter­actions.

## Related literature

For related literature, see: Fang *et al.* (2002 ▶, 2007 ▶); Medlycott & Hanan (2005 ▶, 2006 ▶); Spek (2003 ▶).
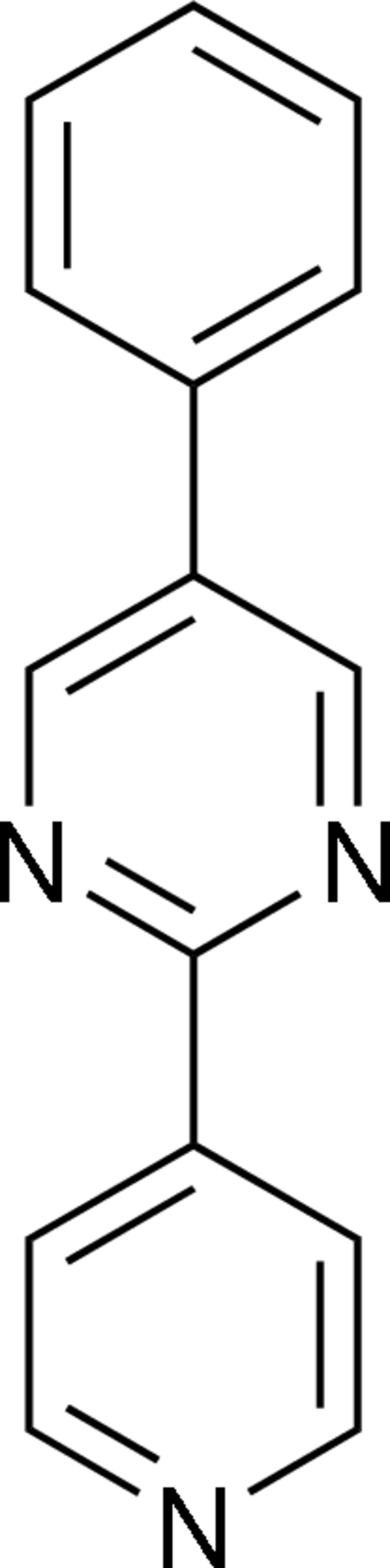


## Experimental

### 

#### Crystal data

C_15_H_11_N_3_
*M* *_r_* = 233.27
Triclinic, 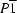

*a* = 9.2813 (5) Å
*b* = 9.3609 (5) Å
*c* = 13.9001 (7) Å
α = 71.462 (2)°
β = 86.957 (2)°
γ = 75.788 (3)°
*V* = 1109.54 (10) Å^3^
*Z* = 4
Cu *K*α radiation
μ = 0.68 mm^−1^
*T* = 100 (2) K
0.40 × 0.38 × 0.08 mm

#### Data collection

Bruker SMART 6000 diffractometer
Absorption correction: multi-scan (*SADABS*; Sheldrick, 1996 ▶) *T*_min_ = 0.734, *T*_max_ = 0.947
15167 measured reflections
3967 independent reflections
3226 reflections with *I* > 2σ(*I*)
*R*_int_ = 0.046

#### Refinement

*R*[*F* ^2^ > 2σ(*F* ^2^)] = 0.044
*wR*(*F* ^2^) = 0.139
*S* = 1.00
3967 reflections
325 parameters
H-atom parameters constrained
Δρ_max_ = 0.27 e Å^−3^
Δρ_min_ = −0.30 e Å^−3^

### 

Data collection: *SMART* (Bruker, 2003 ▶); cell refinement: *SAINT* (Bruker, 1999 ▶); data reduction: *SAINT*; program(s) used to solve structure: *SHELXS97* (Sheldrick, 2008 ▶); program(s) used to refine structure: *SHELXL97* (Sheldrick, 2008 ▶); molecular graphics: *SHELXTL* (Sheldrick, 2008 ▶); software used to prepare material for publication: *UdMX* (local program).

## Supplementary Material

Crystal structure: contains datablocks I, global. DOI: 10.1107/S160053680800398X/lh2593sup1.cif

Structure factors: contains datablocks I. DOI: 10.1107/S160053680800398X/lh2593Isup2.hkl

Additional supplementary materials:  crystallographic information; 3D view; checkCIF report

## Figures and Tables

**Table 1:** Hydrogen-bond geometry (Å, °)

*D*—H⋯*A*	*D*—H	H⋯*A*	*D*⋯*A*	*D*—H⋯*A*
C7—H7⋯N5^i^	0.95	2.55	3.3818 (18)	147
C9—H9⋯N6^ii^	0.95	2.56	3.4027 (18)	148
C22—H22⋯N2^i^	0.95	2.54	3.3784 (18)	147
C24—H24⋯N3^ii^	0.95	2.56	3.4049 (19)	149
C1—H1⋯*Cg*3^iii^	0.95	2.91	3.5925 (15)	129
C4—H4⋯*Cg*3	0.95	2.72	3.4163 (15)	130
C12—H12⋯*Cg*1^i^	0.95	2.89	3.5844 (15)	131
C15—H15⋯*Cg*1^iv^	0.95	2.92	3.5202 (15)	122
C17—H17⋯*Cg*6^v^	0.95	2.84	3.5561 (16)	133
C20—H20⋯*Cg*6^ii^	0.95	2.85	3.5251 (15)	129
C26—H26⋯*Cg*5	0.95	2.86	3.5242 (15)	128
C29—H29⋯*Cg*5^vi^	0.95	2.77	3.4451 (15)	129

Symmetry codes: (i) 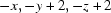; (ii) 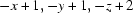; (iii) 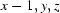; (iv) 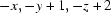; (v) 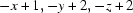; (vi) 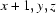. *Cg*1 is the centroid of the N1/C1-C5 ring, *Cg*3 is the centroid of the N4/C16–C20 ring, *Cg*5 is the centroid of the C10–C15 ring and *Cg*6 is the centroid of the C25–C30 ring.

**Fig. 1.**
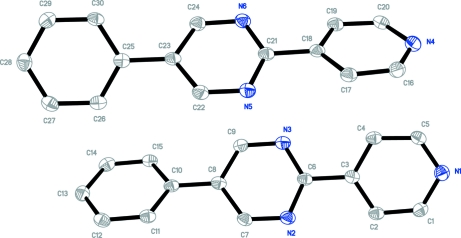
The asymmetric unit with thermal ellipsoids shown at 50% probability levels. H atoms have been omitted.

**Fig. 2.**
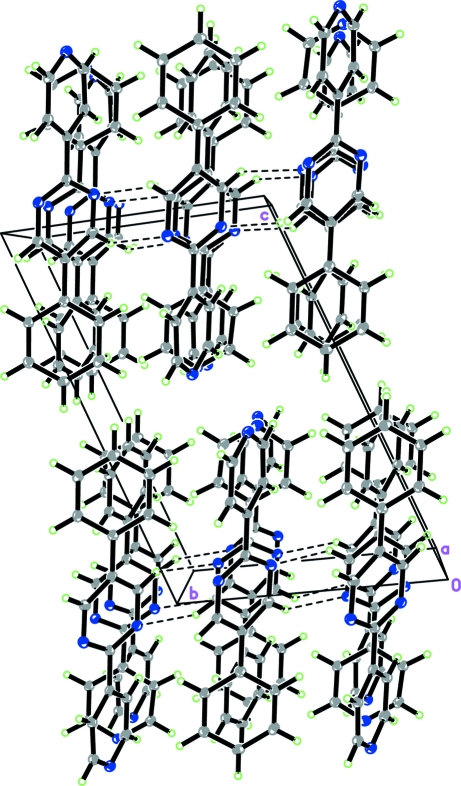
Part of the crystal structure of (3). Hydrogen bonds are shown as dashed lines. Ellipsoids are shown at the 30% probabilty level.

**Fig. 3.**
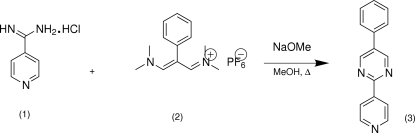
The reaction scheme for the title compound.

## supplementary crystallographic information

### Comment

Ruthenium polypyridyl complexes have long attracted attention due to their exceptional photophysical properties which makes them suitable as chromophores in light-harvesting devices (Medlycott & Hanan, 2005, 2006). Recently, we have reported new pyrimidine-substituted terpyridine ligands and their Ru(II) polypyridyl complexes (Fang *et al.*, 2002, 2007). Introduction of the pyrimidine motif on a terpyridine unit leads to planarization of the system through hydrogen bonds, thus extending pi-delocalization in the acceptor ligand of the metal-to-ligand charge transfer (MLCT) emitting excited-states, which improves the photophysical properties of the complexes. The title compound C_15_H_11_N_3_ (3) [see Fig. 3] was designed for the enhanced π-acceptor character of pyridyl-type ligands for coordination and supramolecular chemistry.

The title compound crystallizes with two molecules per asymmetric unit. The assignment of the nitrogen atoms was confirmed by comparing the observed and expected torsion angles and bond lengths. Ligand (3) is less planar than the terpyridyl analogue (Fang *et al.*, 2007) despite weak intramolecular C—H···lone pair (N) interactions. All non-bonded N···H distances are shorter than 2.75 Å [N2···H2 = 2.57 Å, N3···H4 = 2.57 Å, N5···H17 = 2.62 Å, N6···H19 = 2.51 Å]. The dihedral angles between the phenyl and pyridine rings in each molecule are 53.48 (5)° and 50.80 (5)°. These deviations from planarity, in part, may be influenced by weak intermolecular C—H···N hydrogen bonds connecting molecules into one-dimensional chains and in addition, by the crystal structure being stabilized by weak C—H···π stacking interactions between "head-to-tail" molecules.

### Experimental

4-Pyridylamidine hydrochloride (1) (10,0 g, 63.5 mmol), 2-phenyl-1,3-bis(dimethylamino)trimethinium hexafluorophosphate (2) (1 eq, 22.1 g, 63.5 mmol) and NaOMe (1.2 eq, 4.13 g, 76.5 mmol) were dissolved in anhydrous MeOH (500 ml). The resulting yellow solution was refluxed for 15 h under N_2_. The white solid was isolated by filtration and dried under vacuum to give white shiny micro-crystals (11.0 g, 74%) of pure title compound (3). These crystals were suitable for X-ray diffraction measurements, m.p. 477.8–478.5 K. Anal Calcd for C_15_H_11_N_3_ (233.3): C, 77.23; H, 4.75; N, 18.01. Found: C, 77.04; H, 4.69; N, 17.86.

### Refinement

H atoms were generated geometrically (C—H = 0.95 Å) and were included in the refinement in the riding model approximation; their temperature factors were set to 1.2 times those of the equivalent isotropic temperature factors of the parent site. A final verification of possible voids was performed using the VOID routine of the *PLATON* program (Spek, 2003).

### Crystal data

**Table:**

C_15_H_11_N_3_	*Z* = 4
*M**_r_* = 233.27	*F*(000) = 488
Triclinic, *P*1	*D*_x_ = 1.396 Mg m^−^^3^
Hall symbol: -P 1	Cu *K*α radiation, λ = 1.54178 Å
*a* = 9.2813 (5) Å	Cell parameters from 5655 reflections
*b* = 9.3609 (5) Å	θ = 3.4–68.9°
*c* = 13.9001 (7) Å	µ = 0.68 mm^−^^1^
α = 71.462 (2)°	*T* = 100 K
β = 86.957 (2)°	Block, colourless
γ = 75.788 (3)°	0.40 × 0.38 × 0.08 mm
*V* = 1109.54 (10) Å^3^	

### Data collection

**Table:**

Bruker SMART 6000 diffractometer	3967 independent reflections
Radiation source: Rotating anode	3226 reflections with *I* > 2σ(*I*)
Montel 200 optics	*R*_int_ = 0.046
Detector resolution: 5.5 pixels mm^-1^	θ_max_ = 68.9°, θ_min_ = 3.4°
ω scans	*h* = −11→11
Absorption correction: multi-scan (*SADABS*; Sheldrick, 1996)	*k* = −11→11
*T*_min_ = 0.734, *T*_max_ = 0.947	*l* = −16→16
15167 measured reflections	

### Refinement

**Table:**

Refinement on *F*^2^	Primary atom site location: structure-invariant direct methods
Least-squares matrix: full	Secondary atom site location: difference Fourier map
*R*[*F*^2^ > 2σ(*F*^2^)] = 0.044	Hydrogen site location: inferred from neighbouring sites
*wR*(*F*^2^) = 0.139	H-atom parameters constrained
*S* = 1.00	*w* = 1/[σ^2^(*F*_o_^2^) + (0.0925*P*)^2^ + 0.1892*P*] where *P* = (*F*_o_^2^ + 2*F*_c_^2^)/3
3967 reflections	(Δ/σ)_max_ = 0.001
325 parameters	Δρ_max_ = 0.27 e Å^−^^3^
0 restraints	Δρ_min_ = −0.30 e Å^−^^3^

### Special details

**Table:**

Geometry. All e.s.d.'s (except the e.s.d. in the dihedral angle between two l.s. planes) are estimated using the full covariance matrix. The cell e.s.d.'s are taken into account individually in the estimation of e.s.d.'s in distances, angles and torsion angles; correlations between e.s.d.'s in cell parameters are only used when they are defined by crystal symmetry. An approximate (isotropic) treatment of cell e.s.d.'s is used for estimating e.s.d.'s involving l.s. planes.
Refinement. Refinement of *F*^2^ against ALL reflections. The weighted *R*-factor *wR* and goodness of fit *S* are based on *F*^2^, conventional *R*-factors *R* are based on *F*, with *F* set to zero for negative *F*^2^. The threshold expression of *F*^2^ > σ(*F*^2^) is used only for calculating *R*-factors(gt) *etc*. and is not relevant to the choice of reflections for refinement. *R*-factors based on *F*^2^ are statistically about twice as large as those based on *F*, and *R*- factors based on ALL data will be even larger.

### Fractional atomic coordinates and isotropic or equivalent isotropic displacement parameters (Å^2^)

**Table:**

	*x*	*y*	*z*	*U*_iso_*/*U*_eq_	
N1	−0.09769 (12)	0.44347 (13)	1.42209 (9)	0.0235 (3)	
N2	−0.12326 (12)	0.79515 (13)	1.05916 (8)	0.0215 (3)	
N3	0.10382 (12)	0.60327 (13)	1.06789 (9)	0.0230 (3)	
C28	0.60351 (14)	1.04795 (15)	0.59991 (10)	0.0230 (3)	
H28	0.6235	1.0980	0.5313	0.028*	
C24	0.59612 (14)	0.67217 (15)	0.96148 (10)	0.0211 (3)	
H24	0.6697	0.6196	0.9262	0.025*	
C22	0.40977 (14)	0.89133 (15)	0.96659 (10)	0.0210 (3)	
H22	0.3519	0.9935	0.9349	0.025*	
C1	−0.20867 (14)	0.54005 (15)	1.35782 (10)	0.0222 (3)	
H1	−0.3061	0.5581	1.3835	0.027*	
C2	−0.18962 (14)	0.61497 (15)	1.25624 (10)	0.0206 (3)	
H2	−0.2717	0.6841	1.2144	0.025*	
C3	−0.04783 (14)	0.58719 (14)	1.21624 (10)	0.0188 (3)	
C4	0.06828 (14)	0.48702 (15)	1.28198 (10)	0.0204 (3)	
H4	0.1664	0.4647	1.2580	0.025*	
C5	0.03846 (14)	0.42042 (15)	1.38280 (10)	0.0218 (3)	
H5	0.1193	0.3543	1.4270	0.026*	
C6	−0.02146 (13)	0.66572 (15)	1.10832 (10)	0.0190 (3)	
C7	−0.09684 (14)	0.86669 (15)	0.96277 (10)	0.0207 (3)	
H7	−0.1671	0.9585	0.9265	0.025*	
C8	0.02937 (13)	0.81303 (14)	0.91253 (10)	0.0191 (3)	
C9	0.12654 (14)	0.67759 (15)	0.97125 (10)	0.0224 (3)	
H9	0.2138	0.6359	0.9407	0.027*	
C10	0.05403 (13)	0.89220 (15)	0.80502 (10)	0.0192 (3)	
C11	0.00409 (13)	1.05394 (15)	0.76275 (10)	0.0203 (3)	
H11	−0.0429	1.1139	0.8045	0.024*	
C12	0.02259 (14)	1.12719 (15)	0.66058 (10)	0.0217 (3)	
H12	−0.0111	1.2368	0.6330	0.026*	
C13	0.09030 (14)	1.04032 (16)	0.59868 (10)	0.0223 (3)	
H13	0.1021	1.0903	0.5287	0.027*	
C14	0.14063 (14)	0.88000 (15)	0.63960 (10)	0.0227 (3)	
H14	0.1872	0.8206	0.5974	0.027*	
C15	0.12309 (13)	0.80658 (15)	0.74159 (10)	0.0202 (3)	
H15	0.1582	0.6971	0.7689	0.024*	
C29	0.71673 (14)	0.94102 (15)	0.66453 (10)	0.0221 (3)	
H29	0.8144	0.9178	0.6400	0.027*	
C30	0.68821 (13)	0.86809 (15)	0.76430 (10)	0.0197 (3)	
H30	0.7664	0.7944	0.8077	0.024*	
C25	0.54460 (14)	0.90173 (15)	0.80227 (10)	0.0186 (3)	
C26	0.43120 (14)	1.00916 (15)	0.73613 (10)	0.0203 (3)	
H26	0.3332	1.0326	0.7601	0.024*	
C27	0.46033 (15)	1.08163 (15)	0.63604 (10)	0.0230 (3)	
H27	0.3825	1.1545	0.5919	0.028*	
C23	0.51637 (13)	0.82266 (15)	0.90904 (10)	0.0184 (3)	
N6	0.57446 (11)	0.59835 (13)	1.05838 (8)	0.0210 (3)	
C21	0.46901 (13)	0.67626 (15)	1.10567 (10)	0.0187 (3)	
N5	0.38525 (12)	0.82082 (12)	1.06340 (8)	0.0211 (3)	
C18	0.44441 (13)	0.59364 (15)	1.21314 (10)	0.0186 (3)	
C19	0.49348 (13)	0.43229 (15)	1.25172 (10)	0.0203 (3)	
H19	0.5441	0.3745	1.2097	0.024*	
C20	0.46761 (14)	0.35763 (15)	1.35169 (10)	0.0215 (3)	
H20	0.4998	0.2476	1.3760	0.026*	
N4	0.39970 (12)	0.43088 (13)	1.41673 (8)	0.0238 (3)	
C16	0.35492 (14)	0.58659 (16)	1.37925 (10)	0.0231 (3)	
H16	0.3079	0.6415	1.4238	0.028*	
C17	0.37312 (13)	0.67179 (16)	1.27954 (10)	0.0207 (3)	
H17	0.3377	0.7816	1.2568	0.025*	

### Atomic displacement parameters (Å^2^)

**Table:**

	*U*^11^	*U*^22^	*U*^33^	*U*^12^	*U*^13^	*U*^23^
N1	0.0246 (6)	0.0248 (6)	0.0228 (6)	−0.0064 (5)	−0.0001 (5)	−0.0092 (5)
N2	0.0197 (5)	0.0236 (6)	0.0209 (6)	−0.0007 (5)	−0.0027 (4)	−0.0095 (5)
N3	0.0199 (5)	0.0226 (6)	0.0238 (6)	−0.0003 (5)	0.0001 (5)	−0.0071 (5)
C28	0.0266 (7)	0.0257 (7)	0.0181 (7)	−0.0088 (6)	0.0001 (5)	−0.0070 (6)
C24	0.0192 (6)	0.0235 (7)	0.0211 (7)	−0.0017 (5)	−0.0004 (5)	−0.0104 (6)
C22	0.0198 (6)	0.0187 (6)	0.0229 (7)	−0.0011 (5)	−0.0013 (5)	−0.0068 (5)
C1	0.0192 (6)	0.0252 (7)	0.0250 (7)	−0.0050 (5)	0.0014 (5)	−0.0119 (6)
C2	0.0174 (6)	0.0218 (7)	0.0243 (7)	−0.0032 (5)	−0.0030 (5)	−0.0102 (6)
C3	0.0191 (6)	0.0176 (6)	0.0215 (7)	−0.0033 (5)	−0.0019 (5)	−0.0090 (5)
C4	0.0173 (6)	0.0208 (6)	0.0254 (7)	−0.0033 (5)	−0.0016 (5)	−0.0108 (6)
C5	0.0205 (6)	0.0209 (6)	0.0234 (7)	−0.0029 (5)	−0.0046 (5)	−0.0070 (6)
C6	0.0165 (6)	0.0191 (6)	0.0225 (7)	−0.0019 (5)	−0.0035 (5)	−0.0094 (6)
C7	0.0199 (6)	0.0213 (7)	0.0197 (7)	−0.0001 (5)	−0.0047 (5)	−0.0078 (5)
C8	0.0182 (6)	0.0194 (7)	0.0212 (7)	−0.0025 (5)	−0.0035 (5)	−0.0094 (6)
C9	0.0180 (6)	0.0250 (7)	0.0225 (7)	−0.0012 (5)	0.0010 (5)	−0.0080 (6)
C10	0.0135 (6)	0.0229 (7)	0.0225 (7)	−0.0037 (5)	−0.0025 (5)	−0.0090 (6)
C11	0.0171 (6)	0.0225 (7)	0.0230 (7)	−0.0028 (5)	−0.0029 (5)	−0.0106 (6)
C12	0.0194 (6)	0.0208 (6)	0.0247 (7)	−0.0041 (5)	−0.0049 (5)	−0.0064 (6)
C13	0.0211 (6)	0.0283 (7)	0.0191 (7)	−0.0085 (6)	−0.0010 (5)	−0.0075 (6)
C14	0.0187 (6)	0.0285 (7)	0.0257 (7)	−0.0066 (5)	0.0013 (5)	−0.0145 (6)
C15	0.0160 (6)	0.0193 (6)	0.0256 (7)	−0.0026 (5)	−0.0017 (5)	−0.0085 (6)
C29	0.0200 (6)	0.0279 (7)	0.0228 (7)	−0.0077 (6)	0.0017 (5)	−0.0128 (6)
C30	0.0167 (6)	0.0221 (7)	0.0206 (7)	−0.0016 (5)	−0.0044 (5)	−0.0089 (5)
C25	0.0182 (6)	0.0188 (6)	0.0213 (7)	−0.0040 (5)	−0.0016 (5)	−0.0096 (5)
C26	0.0166 (6)	0.0211 (6)	0.0242 (7)	−0.0030 (5)	−0.0008 (5)	−0.0093 (6)
C27	0.0224 (7)	0.0215 (7)	0.0239 (7)	−0.0032 (5)	−0.0061 (5)	−0.0059 (6)
C23	0.0143 (6)	0.0211 (7)	0.0211 (7)	−0.0030 (5)	−0.0025 (5)	−0.0092 (6)
N6	0.0197 (5)	0.0231 (6)	0.0197 (6)	−0.0008 (5)	−0.0017 (4)	−0.0090 (5)
C21	0.0154 (6)	0.0209 (7)	0.0214 (7)	−0.0029 (5)	−0.0031 (5)	−0.0094 (6)
N5	0.0195 (5)	0.0203 (6)	0.0214 (6)	−0.0014 (5)	0.0000 (4)	−0.0063 (5)
C18	0.0135 (6)	0.0232 (7)	0.0202 (7)	−0.0038 (5)	−0.0024 (5)	−0.0081 (6)
C19	0.0170 (6)	0.0229 (7)	0.0230 (7)	−0.0031 (5)	−0.0034 (5)	−0.0104 (6)
C20	0.0181 (6)	0.0217 (7)	0.0231 (7)	−0.0030 (5)	−0.0051 (5)	−0.0054 (6)
N4	0.0208 (6)	0.0288 (6)	0.0214 (6)	−0.0051 (5)	−0.0024 (5)	−0.0076 (5)
C16	0.0203 (6)	0.0288 (7)	0.0223 (7)	−0.0043 (6)	−0.0002 (5)	−0.0119 (6)
C17	0.0173 (6)	0.0228 (7)	0.0229 (7)	−0.0026 (5)	−0.0022 (5)	−0.0097 (6)

### Geometric parameters (Å, °)

**Table:**

N1—C1	1.3429 (18)	C11—C12	1.3890 (18)
N1—C5	1.3451 (17)	C11—H11	0.95
N2—C7	1.3338 (17)	C12—C13	1.3904 (18)
N2—C6	1.3445 (17)	C12—H12	0.95
N3—C9	1.3344 (17)	C13—C14	1.3911 (19)
N3—C6	1.3474 (16)	C13—H13	0.95
C28—C29	1.3874 (19)	C14—C15	1.3854 (18)
C28—C27	1.3935 (19)	C14—H14	0.95
C28—H28	0.95	C15—H15	0.95
C24—N6	1.3341 (17)	C29—C30	1.3810 (18)
C24—C23	1.3987 (19)	C29—H29	0.95
C24—H24	0.95	C30—C25	1.4068 (18)
C22—N5	1.3321 (17)	C30—H30	0.95
C22—C23	1.4010 (18)	C25—C26	1.3998 (19)
C22—H22	0.95	C25—C23	1.4746 (18)
C1—C2	1.3861 (18)	C26—C27	1.3853 (18)
C1—H1	0.95	C26—H26	0.95
C2—C3	1.3982 (18)	C27—H27	0.95
C2—H2	0.95	N6—C21	1.3446 (16)
C3—C4	1.3924 (19)	C21—N5	1.3442 (17)
C3—C6	1.4829 (18)	C21—C18	1.4825 (18)
C4—C5	1.3847 (18)	C18—C19	1.3962 (19)
C4—H4	0.95	C18—C17	1.3970 (18)
C5—H5	0.95	C19—C20	1.3808 (18)
C7—C8	1.3998 (18)	C19—H19	0.95
C7—H7	0.95	C20—N4	1.3440 (17)
C8—C9	1.3949 (19)	C20—H20	0.95
C8—C10	1.4757 (18)	N4—C16	1.3458 (18)
C9—H9	0.95	C16—C17	1.3871 (19)
C10—C15	1.4027 (18)	C16—H16	0.95
C10—C11	1.4039 (19)	C17—H17	0.95
			
C1—N1—C5	116.27 (12)	C12—C13—C14	119.78 (13)
C7—N2—C6	116.84 (11)	C12—C13—H13	120.1
C9—N3—C6	116.34 (11)	C14—C13—H13	120.1
C29—C28—C27	119.71 (12)	C15—C14—C13	120.31 (12)
C29—C28—H28	120.1	C15—C14—H14	119.8
C27—C28—H28	120.1	C13—C14—H14	119.8
N6—C24—C23	123.58 (11)	C14—C15—C10	120.70 (12)
N6—C24—H24	118.2	C14—C15—H15	119.6
C23—C24—H24	118.2	C10—C15—H15	119.6
N5—C22—C23	123.44 (12)	C30—C29—C28	120.36 (12)
N5—C22—H22	118.3	C30—C29—H29	119.8
C23—C22—H22	118.3	C28—C29—H29	119.8
N1—C1—C2	123.93 (12)	C29—C30—C25	120.69 (12)
N1—C1—H1	118	C29—C30—H30	119.7
C2—C1—H1	118	C25—C30—H30	119.7
C1—C2—C3	119.00 (12)	C26—C25—C30	118.38 (12)
C1—C2—H2	120.5	C26—C25—C23	121.73 (11)
C3—C2—H2	120.5	C30—C25—C23	119.88 (11)
C4—C3—C2	117.68 (12)	C27—C26—C25	120.69 (12)
C4—C3—C6	121.26 (11)	C27—C26—H26	119.7
C2—C3—C6	121.03 (12)	C25—C26—H26	119.7
C5—C4—C3	118.96 (12)	C26—C27—C28	120.18 (12)
C5—C4—H4	120.5	C26—C27—H27	119.9
C3—C4—H4	120.5	C28—C27—H27	119.9
N1—C5—C4	124.14 (12)	C24—C23—C22	114.48 (12)
N1—C5—H5	117.9	C24—C23—C25	122.19 (11)
C4—C5—H5	117.9	C22—C23—C25	123.33 (12)
N2—C6—N3	125.23 (12)	C24—N6—C21	116.46 (11)
N2—C6—C3	117.34 (11)	N5—C21—N6	125.40 (12)
N3—C6—C3	117.42 (11)	N5—C21—C18	118.22 (11)
N2—C7—C8	123.18 (12)	N6—C21—C18	116.37 (11)
N2—C7—H7	118.4	C22—N5—C21	116.63 (11)
C8—C7—H7	118.4	C19—C18—C17	117.45 (12)
C9—C8—C7	114.70 (12)	C19—C18—C21	120.29 (11)
C9—C8—C10	123.20 (11)	C17—C18—C21	122.26 (12)
C7—C8—C10	122.08 (12)	C20—C19—C18	119.28 (12)
N3—C9—C8	123.71 (11)	C20—C19—H19	120.4
N3—C9—H9	118.1	C18—C19—H19	120.4
C8—C9—H9	118.1	N4—C20—C19	124.09 (12)
C15—C10—C11	118.38 (12)	N4—C20—H20	118
C15—C10—C8	120.59 (12)	C19—C20—H20	118
C11—C10—C8	120.99 (11)	C20—N4—C16	116.18 (12)
C12—C11—C10	120.74 (12)	N4—C16—C17	124.03 (12)
C12—C11—H11	119.6	N4—C16—H16	118
C10—C11—H11	119.6	C17—C16—H16	118
C11—C12—C13	120.08 (12)	C16—C17—C18	118.96 (12)
C11—C12—H12	120	C16—C17—H17	120.5
C13—C12—H12	120	C18—C17—H17	120.5
			
C5—N1—C1—C2	−0.23 (18)	C27—C28—C29—C30	−0.06 (19)
N1—C1—C2—C3	1.52 (19)	C28—C29—C30—C25	−0.50 (18)
C1—C2—C3—C4	−1.28 (18)	C29—C30—C25—C26	0.86 (18)
C1—C2—C3—C6	−179.38 (11)	C29—C30—C25—C23	−179.99 (11)
C2—C3—C4—C5	−0.09 (18)	C30—C25—C26—C27	−0.67 (18)
C6—C3—C4—C5	178.00 (11)	C23—C25—C26—C27	−179.80 (11)
C1—N1—C5—C4	−1.28 (19)	C25—C26—C27—C28	0.13 (18)
C3—C4—C5—N1	1.46 (19)	C29—C28—C27—C26	0.25 (19)
C7—N2—C6—N3	−0.15 (18)	N6—C24—C23—C22	−0.22 (18)
C7—N2—C6—C3	178.6 (1)	N6—C24—C23—C25	179.12 (11)
C9—N3—C6—N2	−0.02 (19)	N5—C22—C23—C24	0.14 (18)
C9—N3—C6—C3	−178.77 (11)	N5—C22—C23—C25	−179.20 (11)
C4—C3—C6—N2	−158.60 (12)	C26—C25—C23—C24	148.58 (13)
C2—C3—C6—N2	19.42 (17)	C30—C25—C23—C24	−30.54 (17)
C4—C3—C6—N3	20.25 (17)	C26—C25—C23—C22	−32.13 (18)
C2—C3—C6—N3	−161.73 (12)	C30—C25—C23—C22	148.75 (13)
C6—N2—C7—C8	0.11 (18)	C23—C24—N6—C21	0.22 (18)
N2—C7—C8—C9	0.08 (18)	C24—N6—C21—N5	−0.14 (18)
N2—C7—C8—C10	178.41 (11)	C24—N6—C21—C18	179.6 (1)
C6—N3—C9—C8	0.23 (19)	C23—C22—N5—C21	−0.07 (18)
C7—C8—C9—N3	−0.26 (19)	N6—C21—N5—C22	0.07 (19)
C10—C8—C9—N3	−178.57 (11)	C18—C21—N5—C22	−179.67 (11)
C9—C8—C10—C15	33.47 (18)	N5—C21—C18—C19	160.88 (12)
C7—C8—C10—C15	−144.72 (13)	N6—C21—C18—C19	−18.89 (17)
C9—C8—C10—C11	−148.99 (13)	N5—C21—C18—C17	−19.43 (18)
C7—C8—C10—C11	32.82 (18)	N6—C21—C18—C17	160.81 (12)
C15—C10—C11—C12	0.05 (18)	C17—C18—C19—C20	1.14 (18)
C8—C10—C11—C12	−177.54 (11)	C21—C18—C19—C20	−179.15 (10)
C10—C11—C12—C13	0.43 (18)	C18—C19—C20—N4	−1.45 (19)
C11—C12—C13—C14	−0.57 (19)	C19—C20—N4—C16	0.40 (18)
C12—C13—C14—C15	0.24 (19)	C20—N4—C16—C17	0.95 (19)
C13—C14—C15—C10	0.25 (18)	N4—C16—C17—C18	−1.19 (19)
C11—C10—C15—C14	−0.39 (18)	C19—C18—C17—C16	0.08 (18)
C8—C10—C15—C14	177.20 (11)	C21—C18—C17—C16	−179.62 (11)

### Hydrogen-bond geometry (Å, °)

**Table:**

*D*—H···*A*	*D*—H	H···*A*	*D*···*A*	*D*—H···*A*
C7—H7···N5^i^	0.95	2.55	3.3818 (18)	147
C9—H9···N6^ii^	0.95	2.56	3.4027 (18)	148
C22—H22···N2^i^	0.95	2.54	3.3784 (18)	147
C24—H24···N3^ii^	0.95	2.56	3.4049 (19)	149
C1—H1···Cg3^iii^	0.95	2.91	3.5925 (15)	129.
C4—H4···Cg3	0.95	2.72	3.4163 (15)	130.
C12—H12···Cg1^i^	0.95	2.89	3.5844 (15)	131.
C15—H15···Cg1^iv^	0.95	2.92	3.5202 (15)	122.
C17—H17···Cg6^v^	0.95	2.84	3.5561 (16)	133.
C20—H20···Cg6^ii^	0.95	2.85	3.5251 (15)	129.
C26—H26···Cg5	0.95	2.86	3.5242 (15)	128.
C29—H29···Cg5^vi^	0.95	2.77	3.4451 (15)	129.

Symmetry codes: (i) −*x*, −*y*+2, −*z*+2; (ii) −*x*+1, −*y*+1, −*z*+2; (iii) *x*−1, *y*, *z*;  (iv) −*x*, −*y*+1, −*z*+2; (v) −*x*+1, −*y*+2, −*z*+2; (vi) *x*+1, *y*, *z*.
